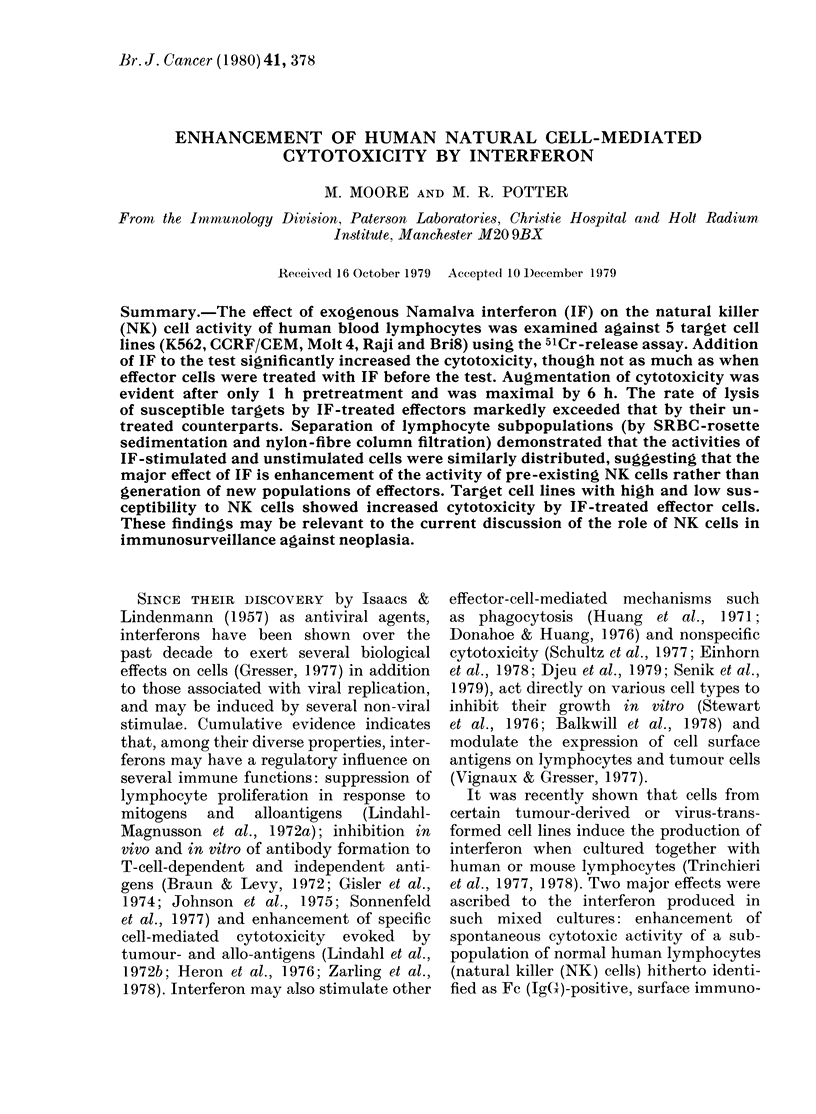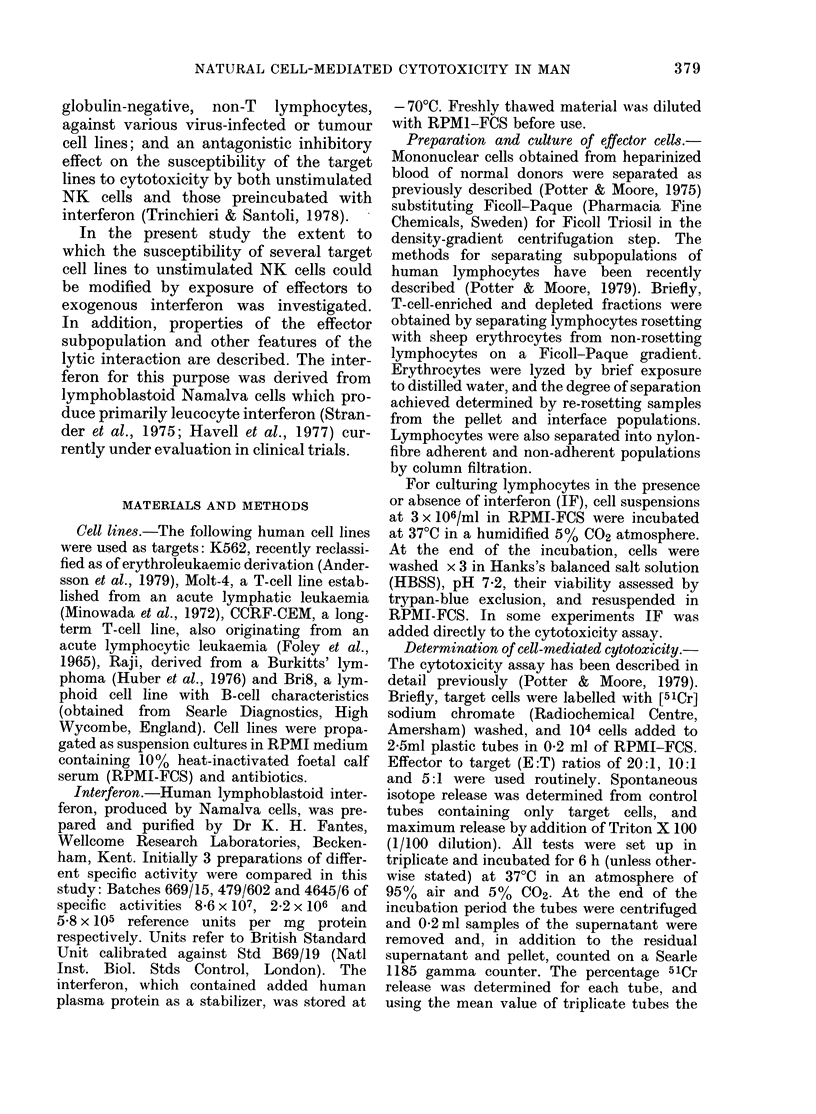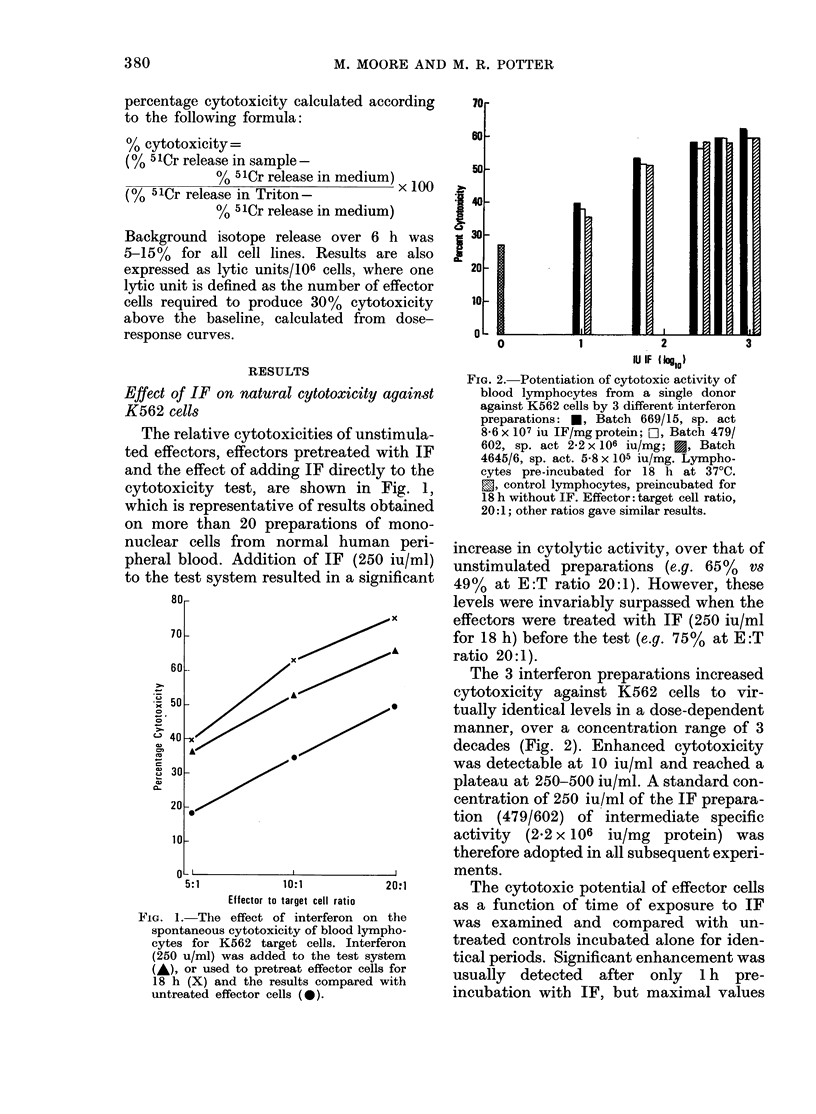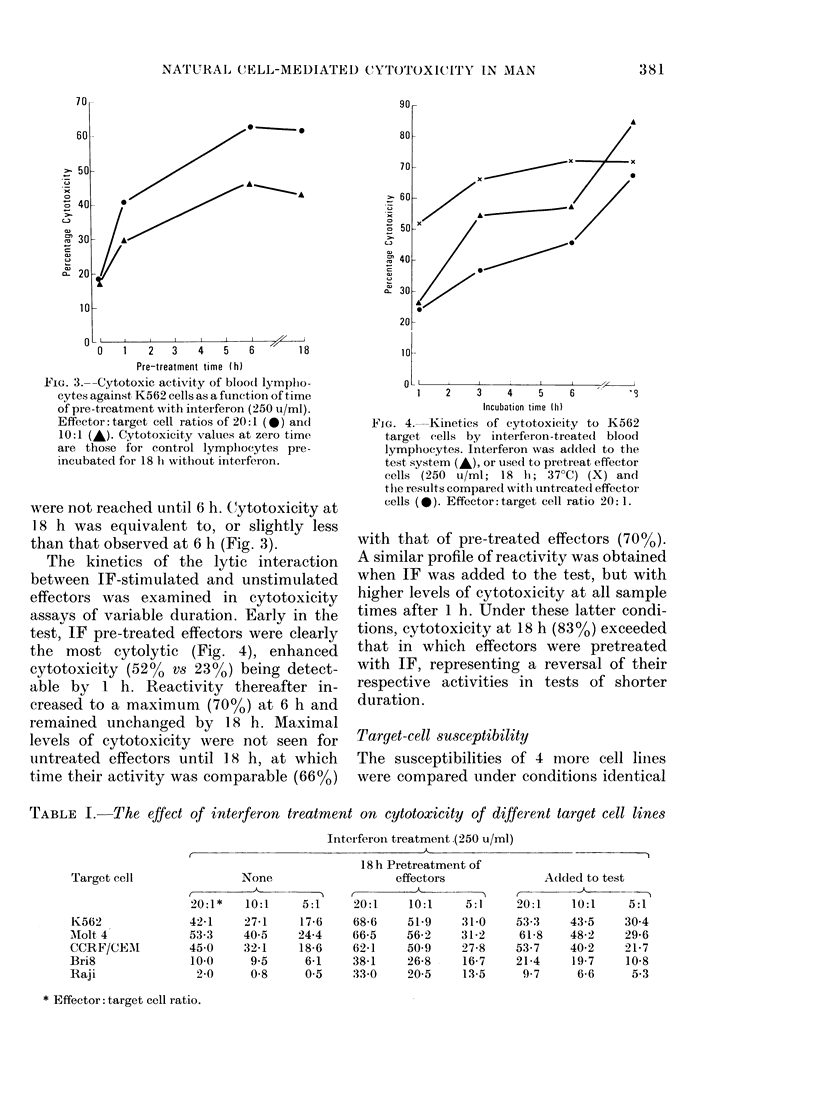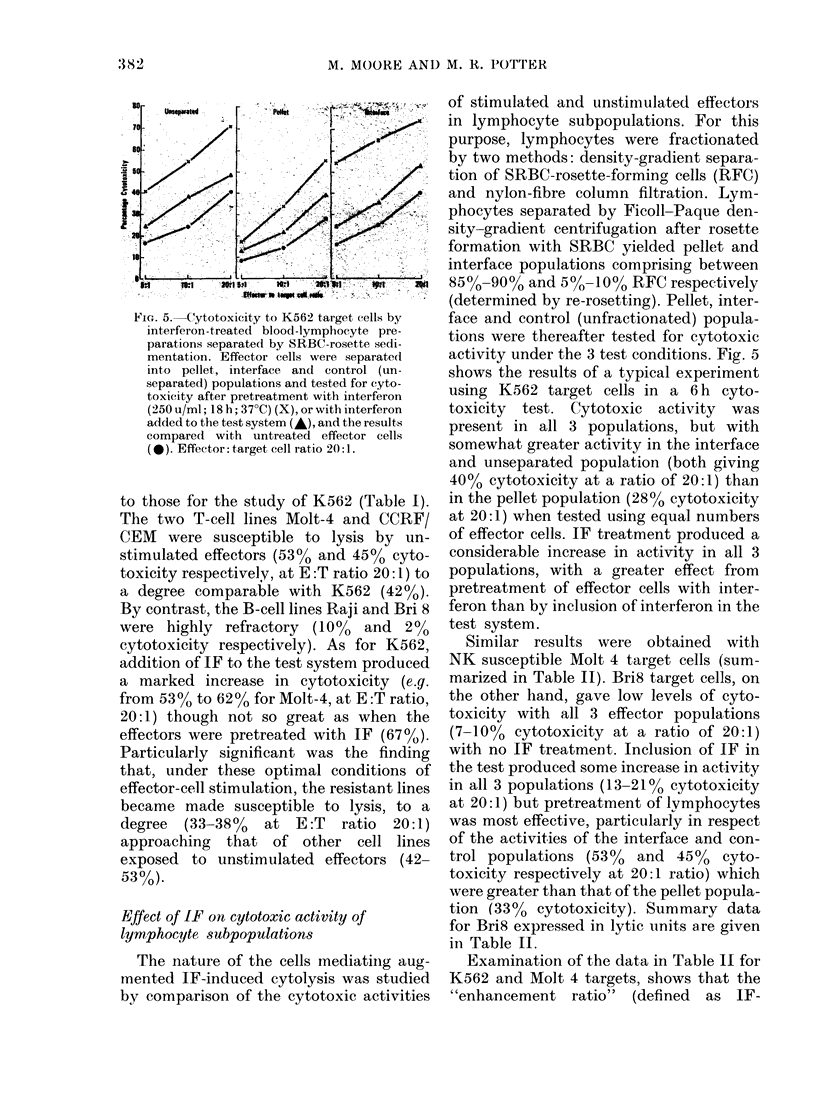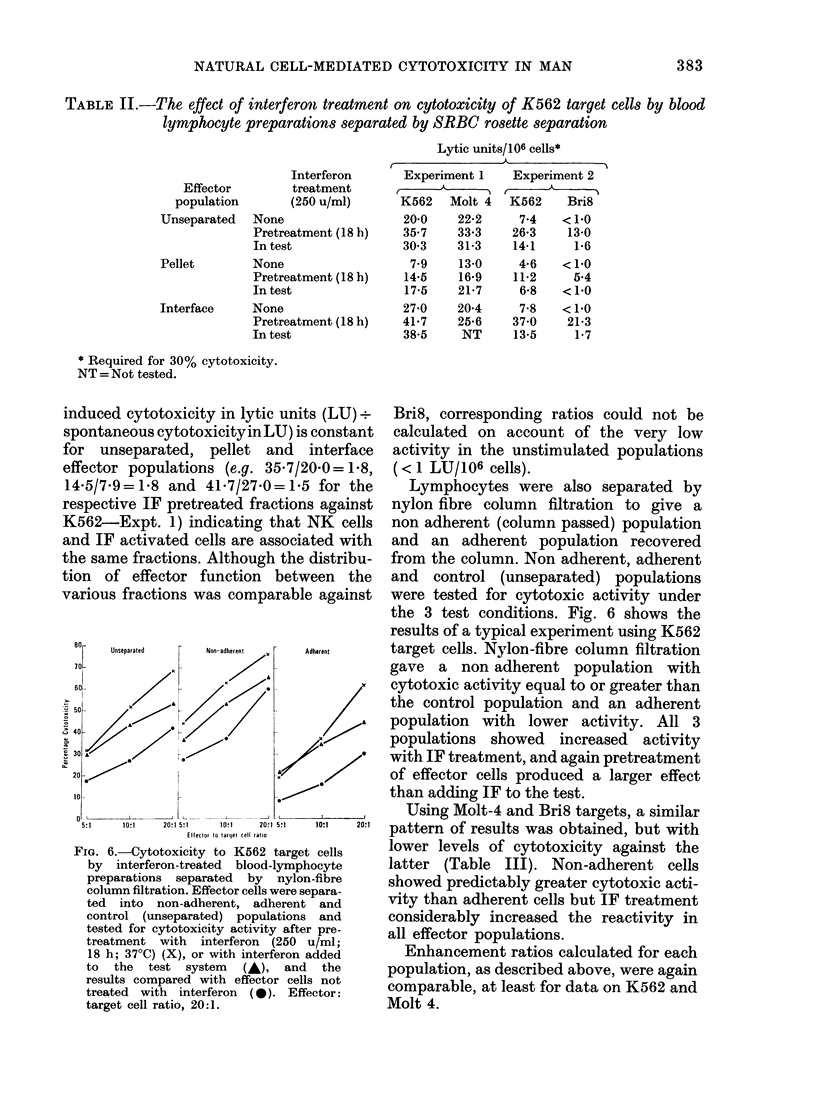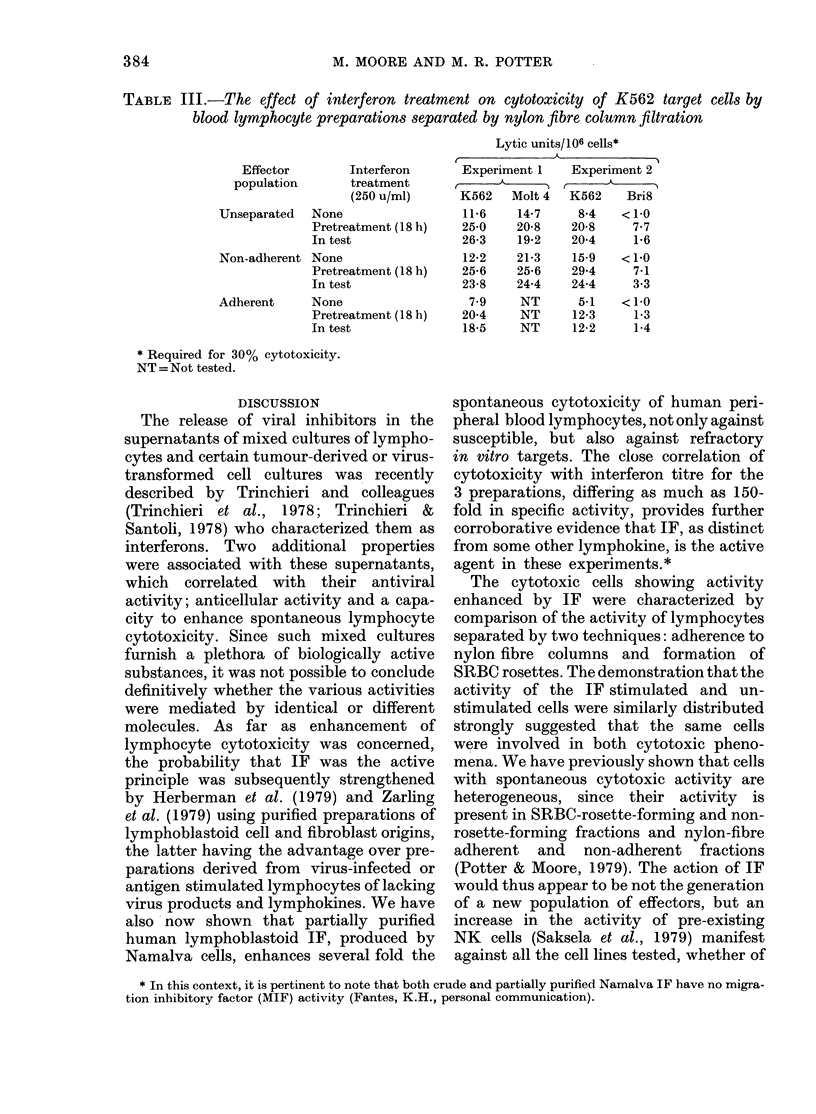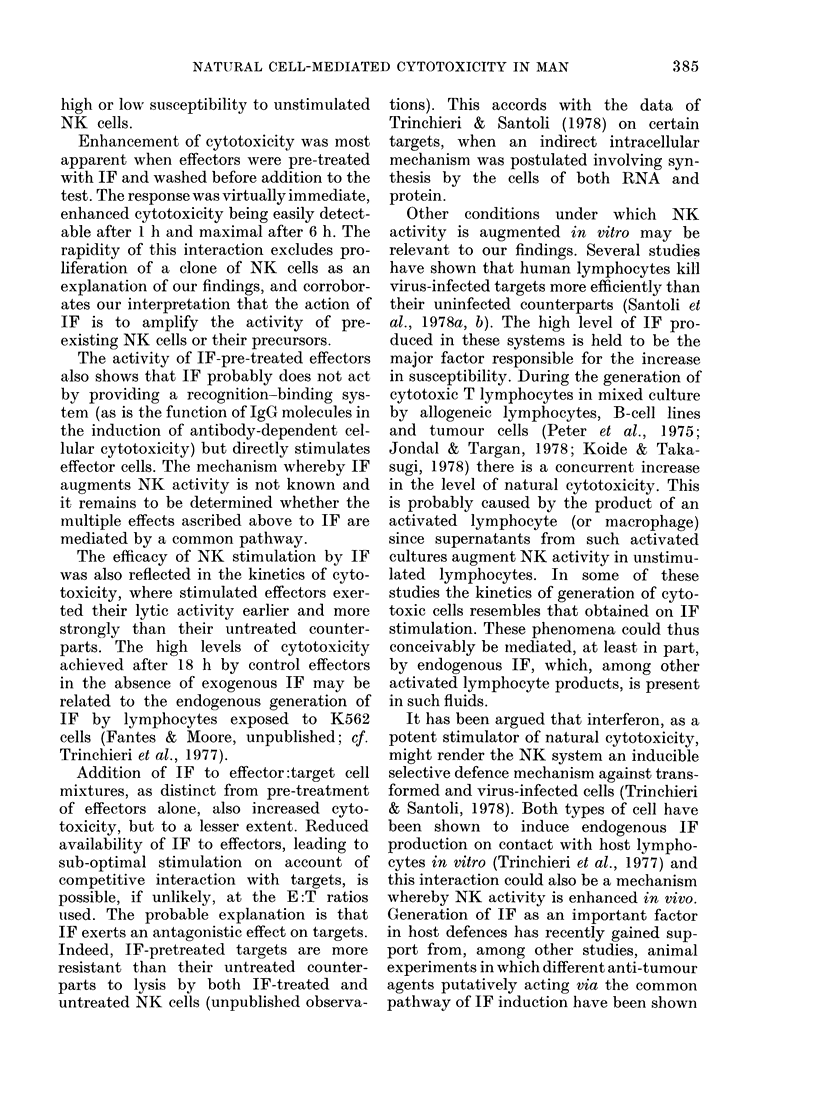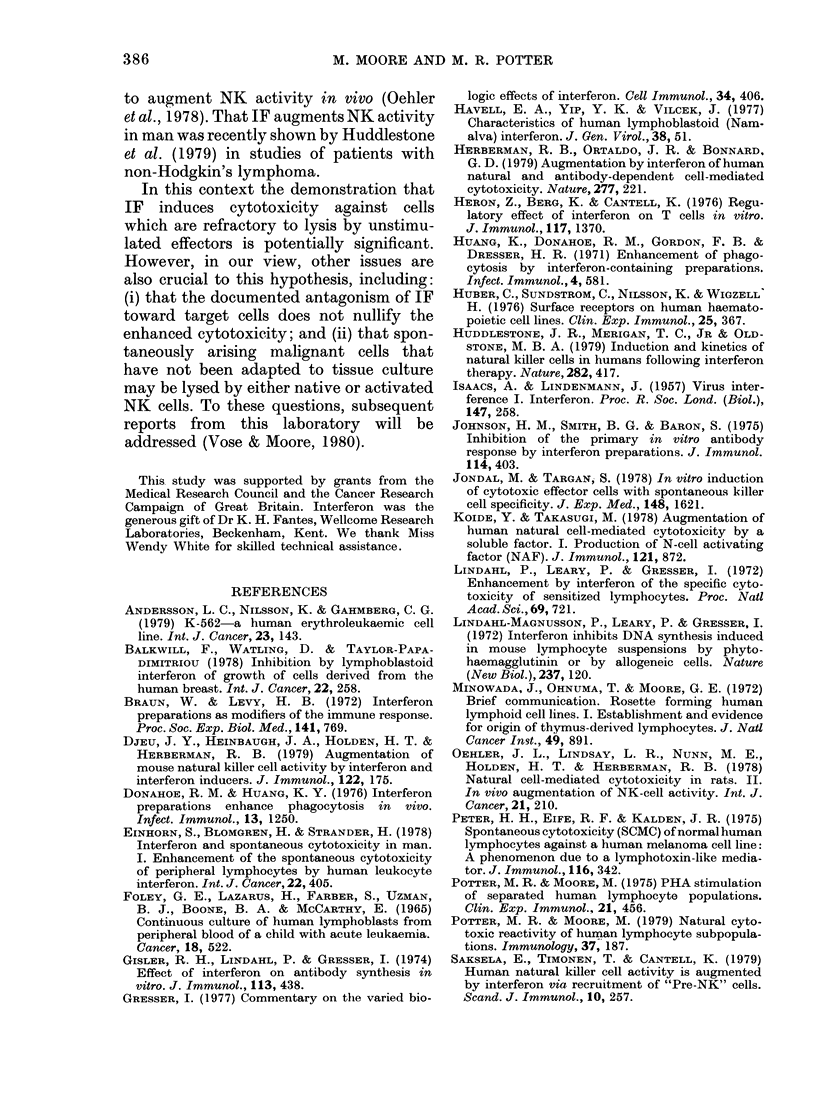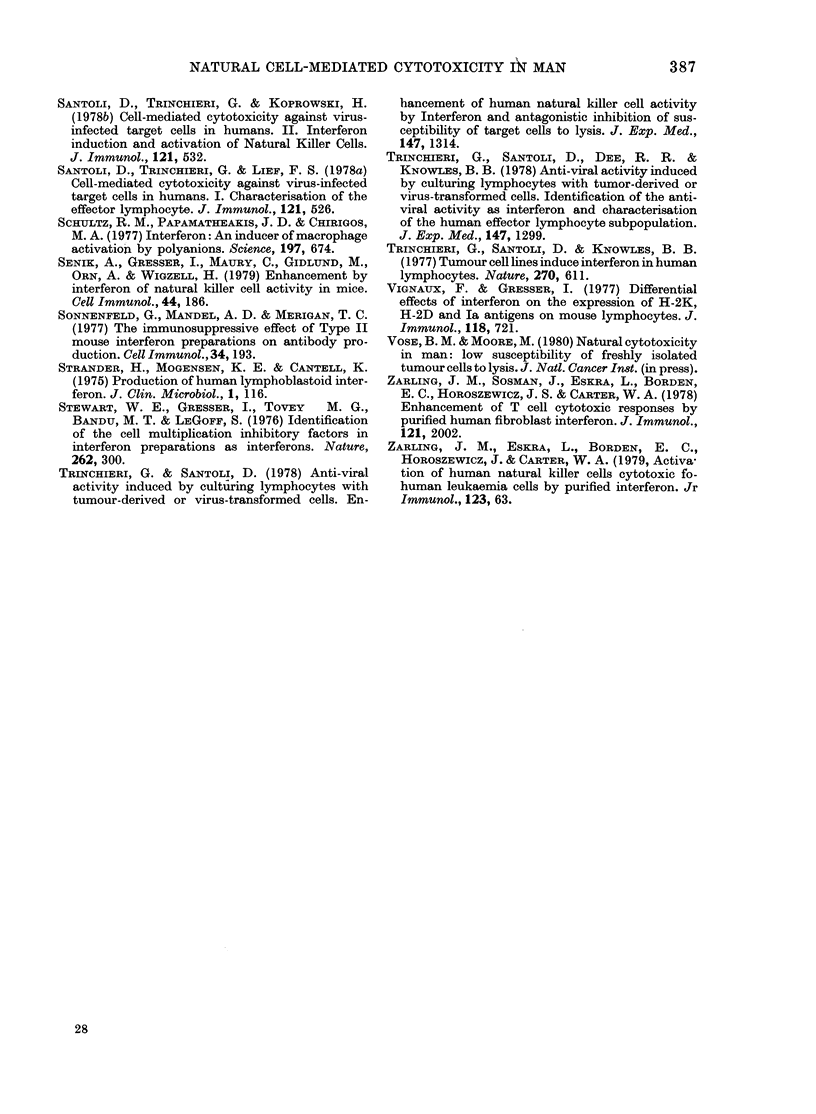# Enhancement of human natural cell-mediated cytotoxicity by interferon.

**DOI:** 10.1038/bjc.1980.61

**Published:** 1980-03

**Authors:** M. Moore, M. R. Potter

## Abstract

The effect of exogenous Namalva interferon (IF) on the natural killer (NK) cell activity of human blood lymphocytes was examined against 5 target cell lines (K562, CCRF/CEM, Molt 4, Raji and Bri8) using the 51Cr-release assay. Addition of IF to the test significantly increased the cytotoxicity, though not as much as when effector cells were treated with IF before the test. Augmentation of cytotoxicity was evident after only 1 h pretreatment and was maximal by 6 h. The rate of lysis of susceptible targets by IF-treated effectors markedly exceeded that by their untreated counterparts. Separation of lymphocyte subpopulations (by SRBC-rosette sedimentation and nylon-fibre column filtration) demonstrated that the activities of IF-stimulated and unstimulated cells were similarly distributed, suggesting that the major effect of IF is enhancement of the activity of pre-existing NK cells rather than generation of new populations of effectors. Target cell lines with high and low susceptibility to NK cells showed increased cytotoxicity by IF-treated effector cells. These findings may be relevant to the current discussion of the role of NK cells in immunosurveillance against neoplasia.


*Br. J. Cancer* (1980) **41**, 378

### ENHANCEMENT OF HUMAN NATURAL CELL-MEDIATED CYTOTOXICITY BY INTERFERON

M. MOORE AND M. R. POTTER

*From the Immunology Division, Paterson Laboratories, Christie Hospital and Holt Radium Institute, Manchester M20 9BX*

Received 16 October 1979 Accepted 10 December 1979

**Summary.—The effect of exogenous Namalva interferon (IF) on the natural killer (NK) cell activity of human blood lymphocytes was examined against 5 target cell lines (K562, CCRF/CEM, Molt 4, Raji and Bri8) using the $^{51}$Cr-release assay. Addition of IF to the test significantly increased the cytotoxicity, though not as much as when effector cells were treated with IF before the test. Augmentation of cytotoxicity was evident after only 1 h pretreatment and was maximal by 6 h. The rate of lysis of susceptible targets by IF-treated effectors markedly exceeded that by their untreated counterparts. Separation of lymphocyte subpopulations (by SRBC-rosette sedimentation and nylon-fibre column filtration) demonstrated that the activities of IF-stimulated and unstimulated cells were similarly distributed, suggesting that the major effect of IF is enhancement of the activity of pre-existing NK cells rather than generation of new populations of effectors. Target cell lines with high and low susceptibility to NK cells showed increased cytotoxicity by IF-treated effector cells. These findings may be relevant to the current discussion of the role of NK cells in immunosurveillance against neoplasia.**

SINCE THEIR DISCOVERY by Isaacs & Lindenmann (1957) as antiviral agents, interferons have been shown over the past decade to exert several biological effects on cells (Gresser, 1977) in addition to those associated with viral replication, and may be induced by several non-viral stimulae. Cumulative evidence indicates that, among their diverse properties, interferons may have a regulatory influence on several immune functions: suppression of lymphocyte proliferation in response to mitogens and alloantigens (Lindahl-Magnusson *et al.*, 1972*a*); inhibition *in vivo* and *in vitro* of antibody formation to T-cell-dependent and independent antigens (Braun & Levy, 1972; Gisler *et al.*, 1974; Johnson *et al.*, 1975; Sonnenfeld *et al.*, 1977) and enhancement of specific cell-mediated cytotoxicity evoked by tumour- and allo-antigens (Lindahl *et al.*, 1972*b*; Heron *et al.*, 1976; Zarling *et al.*, 1978). Interferon may also stimulate other effector-cell-mediated mechanisms such as phagocytosis (Huang *et al.*, 1971; Donahoe & Huang, 1976) and nonspecific cytotoxicity (Schultz *et al.*, 1977; Einhorn *et al.*, 1978; Djeu *et al.*, 1979; Senik *et al.*, 1979), act directly on various cell types to inhibit their growth *in vitro* (Stewart *et al.*, 1976; Balkwill *et al.*, 1978) and modulate the expression of cell surface antigens on lymphocytes and tumour cells (Vignaux & Gresser, 1977).

It was recently shown that cells from certain tumour-derived or virus-transformed cell lines induce the production of interferon when cultured together with human or mouse lymphocytes (Trinchieri *et al.*, 1977, 1978). Two major effects were ascribed to the interferon produced in such mixed cultures: enhancement of spontaneous cytotoxic activity of a subpopulation of normal human lymphocytes (natural killer (NK) cells) hitherto identified as Fc (IgG)-positive, surface immunoglobulin-negative, non-T lymphocytes, against various virus-infected or tumour cell lines; and an antagonistic inhibitory effect on the susceptibility of the target lines to cytotoxicity by both unstimulated NK cells and those preincubated with interferon (Trinchieri & Santoli, 1978).

In the present study the extent to which the susceptibility of several target cell lines to unstimulated NK cells could be modified by exposure of effectors to exogenous interferon was investigated. In addition, properties of the effector subpopulation and other features of the lytic interaction are described. The interferon for this purpose was derived from lymphoblastoid Namalva cells which produce primarily leucocyte interferon (Strander *et al.*, 1975; Havell *et al.*, 1977) currently under evaluation in clinical trials.

### MATERIALS AND METHODS

*Cell lines.*—The following human cell lines were used as targets: K562, recently reclassified as of erythroleukaemic derivation (Andersson *et al.*, 1979), Molt-4, a T-cell line established from an acute lymphatic leukaemia (Minowada *et al.*, 1972), CCRF-CEM, a long-term T-cell line, also originating from an acute lymphocytic leukaemia (Foley *et al.*, 1965), Raji, derived from a Burkitts' lymphoma (Huber *et al.*, 1976) and Bri8, a lymphoid cell line with B-cell characteristics (obtained from Searle Diagnostics, High Wycombe, England). Cell lines were propagated as suspension cultures in RPMI medium containing 10% heat-inactivated foetal calf serum (RPMI-FCS) and antibiotics.

*Interferon.*—Human lymphoblastoid interferon, produced by Namalva cells, was prepared and purified by Dr K. H. Fantes, Wellcome Research Laboratories, Beckenham, Kent. Initially 3 preparations of different specific activity were compared in this study: Batches 669/15, 479/602 and 4645/6 of specific activities $8{\cdot}6\times10^7$, $2{\cdot}2\times10^6$ and $5{\cdot}8\times10^5$ reference units per mg protein respectively. Units refer to British Standard Unit calibrated against Std B69/19 (Natl Inst. Biol. Stds Control, London). The interferon, which contained added human plasma protein as a stabilizer, was stored at −70°C. Freshly thawed material was diluted with RPM1–FCS before use.

*Preparation and culture of effector cells.*—Mononuclear cells obtained from heparinized blood of normal donors were separated as previously described (Potter & Moore, 1975) substituting Ficoll–Paque (Pharmacia Fine Chemicals, Sweden) for Ficoll Triosil in the density-gradient centrifugation step. The methods for separating subpopulations of human lymphocytes have been recently described (Potter & Moore, 1979). Briefly, T-cell-enriched and depleted fractions were obtained by separating lymphocytes rosetting with sheep erythrocytes from non-rosetting lymphocytes on a Ficoll–Paque gradient. Erythrocytes were lyzed by brief exposure to distilled water, and the degree of separation achieved determined by re-rosetting samples from the pellet and interface populations. Lymphocytes were also separated into nylon-fibre adherent and non-adherent populations by column filtration.

For culturing lymphocytes in the presence or absence of interferon (IF), cell suspensions at $3\times10^6$/ml in RPMI-FCS were incubated at 37°C in a humidified 5% $CO_2$ atmosphere. At the end of the incubation, cells were washed ×3 in Hanks's balanced salt solution (HBSS), pH 7·2, their viability assessed by trypan-blue exclusion, and resuspended in RPMI-FCS. In some experiments IF was added directly to the cytotoxicity assay.

*Determination of cell-mediated cytotoxicity.*—The cytotoxicity assay has been described in detail previously (Potter & Moore, 1979). Briefly, target cells were labelled with [$^{51}$Cr] sodium chromate (Radiochemical Centre, Amersham) washed, and $10^4$ cells added to 2·5ml plastic tubes in 0·2 ml of RPMI–FCS. Effector to target (E:T) ratios of 20:1, 10:1 and 5:1 were used routinely. Spontaneous isotope release was determined from control tubes containing only target cells, and maximum release by addition of Triton X 100 (1/100 dilution). All tests were set up in triplicate and incubated for 6 h (unless otherwise stated) at 37°C in an atmosphere of 95% air and 5% $CO_2$. At the end of the incubation period the tubes were centrifuged and 0·2 ml samples of the supernatant were removed and, in addition to the residual supernatant and pellet, counted on a Searle 1185 gamma counter. The percentage $^{51}$Cr release was determined for each tube, and using the mean value of triplicate tubes the percentage cytotoxicity calculated according to the following formula:

$$\% \text{ cytotoxicity} = \frac{(\% \ ^{51}\text{Cr release in sample} - \% \ ^{51}\text{Cr release in medium})}{(\% \ ^{51}\text{Cr release in Triton} - \% \ ^{51}\text{Cr release in medium})} \times 100$$

Background isotope release over 6 h was 5–15% for all cell lines. Results are also expressed as lytic units/$10^6$ cells, where one lytic unit is defined as the number of effector cells required to produce 30% cytotoxicity above the baseline, calculated from dose–response curves.

### RESULTS

### *Effect of IF on natural cytotoxicity against K562 cells*

The relative cytotoxicities of unstimulated effectors, effectors pretreated with IF and the effect of adding IF directly to the cytotoxicity test, are shown in Fig. 1, which is representative of results obtained on more than 20 preparations of mononuclear cells from normal human peripheral blood. Addition of IF (250 iu/ml) to the test system resulted in a significant increase in cytolytic activity, over that of unstimulated preparations (*e.g.* 65% *vs* 49% at E:T ratio 20:1). However, these levels were invariably surpassed when the effectors were treated with IF (250 iu/ml for 18 h) before the test (*e.g.* 75% at E:T ratio 20:1).

FIG. 1.—The effect of interferon on the spontaneous cytotoxicity of blood lymphocytes for K562 target cells. Interferon (250 u/ml) was added to the test system (▲), or used to pretreat effector cells for 18 h (X) and the results compared with untreated effector cells (●).

FIG. 2.—Potentiation of cytotoxic activity of blood lymphocytes from a single donor against K562 cells by 3 different interferon preparations: ■, Batch 669/15, sp. act $8 \cdot 6 \times 10^7$ iu IF/mg protein; □, Batch 479/602, sp. act $2 \cdot 2 \times 10^6$ iu/mg; ▨, Batch 4645/6, sp. act. $5 \cdot 8 \times 10^5$ iu/mg. Lymphocytes pre-incubated for 18 h at 37°C. ▩, control lymphocytes, preincubated for 18 h without IF. Effector: target cell ratio, 20:1; other ratios gave similar results.

The 3 interferon preparations increased cytotoxicity against K562 cells to virtually identical levels in a dose-dependent manner, over a concentration range of 3 decades (Fig. 2). Enhanced cytotoxicity was detectable at 10 iu/ml and reached a plateau at 250–500 iu/ml. A standard concentration of 250 iu/ml of the IF preparation (479/602) of intermediate specific activity ($2 \cdot 2 \times 10^6$ iu/mg protein) was therefore adopted in all subsequent experiments.

The cytotoxic potential of effector cells as a function of time of exposure to IF was examined and compared with untreated controls incubated alone for identical periods. Significant enhancement was usually detected after only 1 h preincubation with IF, but maximal values

FIG. 3.—Cytotoxic activity of blood lymphocytes against K562 cells as a function of time of pre-treatment with interferon (250 u/ml). Effector: target cell ratios of 20:1 (●) and 10:1 (▲). Cytotoxicity values at zero time are those for control lymphocytes pre-incubated for 18 h without interferon.

FIG. 4.—Kinetics of cytotoxicity to K562 target cells by interferon-treated blood lymphocytes. Interferon was added to the test system (▲), or used to pretreat effector cells (250 u/ml; 18 h; 37°C) (X) and the results compared with untreated effector cells (●). Effector: target cell ratio 20:1.

were not reached until 6 h. Cytotoxicity at 18 h was equivalent to, or slightly less than that observed at 6 h (Fig. 3).

The kinetics of the lytic interaction between IF-stimulated and unstimulated effectors was examined in cytotoxicity assays of variable duration. Early in the test, IF pre-treated effectors were clearly the most cytolytic (Fig. 4), enhanced cytotoxicity (52% *vs* 23%) being detectable by 1 h. Reactivity thereafter increased to a maximum (70%) at 6 h and remained unchanged by 18 h. Maximal levels of cytotoxicity were not seen for untreated effectors until 18 h, at which time their activity was comparable (66%) with that of pre-treated effectors (70%). A similar profile of reactivity was obtained when IF was added to the test, but with higher levels of cytotoxicity at all sample times after 1 h. Under these latter conditions, cytotoxicity at 18 h (83%) exceeded that in which effectors were pretreated with IF, representing a reversal of their respective activities in tests of shorter duration.

### *Target-cell susceptibility*

The susceptibilities of 4 more cell lines were compared under conditions identical

TABLE I.—*The effect of interferon treatment on cytotoxicity of different target cell lines*

| Target cell | Interferon treatment (250 u/ml): None | | | 18 h Pretreatment of effectors | | | Added to test | | |
|---|---|---|---|---|---|---|---|---|---|
| | 20:1* | 10:1 | 5:1 | 20:1 | 10:1 | 5:1 | 20:1 | 10:1 | 5:1 |
| K562 | 42·1 | 27·1 | 17·6 | 68·6 | 51·9 | 31·0 | 53·3 | 43·5 | 30·4 |
| Molt 4 | 53·3 | 40·5 | 24·4 | 66·5 | 56·2 | 31·2 | 61·8 | 48·2 | 29·6 |
| CCRF/CEM | 45·0 | 32·1 | 18·6 | 62·1 | 50·9 | 27·8 | 53·7 | 40·2 | 21·7 |
| Bri8 | 10·0 | 9·5 | 6·1 | 38·1 | 26·8 | 16·7 | 21·4 | 19·7 | 10·8 |
| Raji | 2·0 | 0·8 | 0·5 | 33·0 | 20·5 | 13·5 | 9·7 | 6·6 | 5·3 |

* Effector: target cell ratio.

FIG. 5.—Cytotoxicity to K562 target cells by interferon-treated blood-lymphocyte preparations separated by SRBC-rosette sedimentation. Effector cells were separated into pellet, interface and control (unseparated) populations and tested for cytotoxicity after pretreatment with interferon (250 u/ml; 18 h; 37°C) (X), or with interferon added to the test system (▲), and the results compared with untreated effector cells (●). Effector: target cell ratio 20:1.

to those for the study of K562 (Table I). The two T-cell lines Molt-4 and CCRF/CEM were susceptible to lysis by unstimulated effectors (53% and 45% cytotoxicity respectively, at E:T ratio 20:1) to a degree comparable with K562 (42%). By contrast, the B-cell lines Raji and Bri 8 were highly refractory (10% and 2% cytotoxicity respectively). As for K562, addition of IF to the test system produced a marked increase in cytotoxicity (*e.g.* from 53% to 62% for Molt-4, at E:T ratio, 20:1) though not so great as when the effectors were pretreated with IF (67%). Particularly significant was the finding that, under these optimal conditions of effector-cell stimulation, the resistant lines became made susceptible to lysis, to a degree (33–38% at E:T ratio 20:1) approaching that of other cell lines exposed to unstimulated effectors (42–53%).

*Effect of IF on cytotoxic activity of lymphocyte subpopulations*

The nature of the cells mediating augmented IF-induced cytolysis was studied by comparison of the cytotoxic activities of stimulated and unstimulated effectors in lymphocyte subpopulations. For this purpose, lymphocytes were fractionated by two methods: density-gradient separation of SRBC-rosette-forming cells (RFC) and nylon-fibre column filtration. Lymphocytes separated by Ficoll–Paque density–gradient centrifugation after rosette formation with SRBC yielded pellet and interface populations comprising between 85%–90% and 5%–10% RFC respectively (determined by re-rosetting). Pellet, interface and control (unfractionated) populations were thereafter tested for cytotoxic activity under the 3 test conditions. Fig. 5 shows the results of a typical experiment using K562 target cells in a 6 h cytotoxicity test. Cytotoxic activity was present in all 3 populations, but with somewhat greater activity in the interface and unseparated population (both giving 40% cytotoxicity at a ratio of 20:1) than in the pellet population (28% cytotoxicity at 20:1) when tested using equal numbers of effector cells. IF treatment produced a considerable increase in activity in all 3 populations, with a greater effect from pretreatment of effector cells with interferon than by inclusion of interferon in the test system.

Similar results were obtained with NK susceptible Molt 4 target cells (summarized in Table II). Bri8 target cells, on the other hand, gave low levels of cytotoxicity with all 3 effector populations (7–10% cytotoxicity at a ratio of 20:1) with no IF treatment. Inclusion of IF in the test produced some increase in activity in all 3 populations (13–21% cytotoxicity at 20:1) but pretreatment of lymphocytes was most effective, particularly in respect of the activities of the interface and control populations (53% and 45% cytotoxicity respectively at 20:1 ratio) which were greater than that of the pellet population (33% cytotoxicity). Summary data for Bri8 expressed in lytic units are given in Table II.

Examination of the data in Table II for K562 and Molt 4 targets, shows that the "enhancement ratio" (defined as IF-

TABLE II.—*The effect of interferon treatment on cytotoxicity of K562 target cells by blood lymphocyte preparations separated by SRBC rosette separation*

| Effector population | Interferon treatment (250 u/ml) | Lytic units/$10^6$ cells* Experiment 1 K562 | Molt 4 | Experiment 2 K562 | Bri8 |
|---|---|---|---|---|---|
| Unseparated | None | 20·0 | 22·2 | 7·4 | <1·0 |
| | Pretreatment (18 h) | 35·7 | 33·3 | 26·3 | 13·0 |
| | In test | 30·3 | 31·3 | 14·1 | 1·6 |
| Pellet | None | 7·9 | 13·0 | 4·6 | <1·0 |
| | Pretreatment (18 h) | 14·5 | 16·9 | 11·2 | 5·4 |
| | In test | 17·5 | 21·7 | 6·8 | <1·0 |
| Interface | None | 27·0 | 20·4 | 7·8 | <1·0 |
| | Pretreatment (18 h) | 41·7 | 25·6 | 37·0 | 21·3 |
| | In test | 38·5 | NT | 13·5 | 1·7 |

* Required for 30% cytotoxicity.
NT = Not tested.

induced cytotoxicity in lytic units (LU) ÷ spontaneous cytotoxicity in LU) is constant for unseparated, pellet and interface effector populations (*e.g.* 35·7/20·0 = 1·8, 14·5/7·9 = 1·8 and 41·7/27·0 = 1·5 for the respective IF pretreated fractions against K562—Expt. 1) indicating that NK cells and IF activated cells are associated with the same fractions. Although the distribution of effector function between the various fractions was comparable against Bri8, corresponding ratios could not be calculated on account of the very low activity in the unstimulated populations (< 1 LU/$10^6$ cells).

FIG. 6.—Cytotoxicity to K562 target cells by interferon-treated blood-lymphocyte preparations separated by nylon-fibre column filtration. Effector cells were separated into non-adherent, adherent and control (unseparated) populations and tested for cytotoxicity activity after pretreatment with interferon (250 u/ml; 18 h; 37°C) (X), or with interferon added to the test system (▲), and the results compared with effector cells not treated with interferon (●). Effector: target cell ratio, 20:1.

Lymphocytes were also separated by nylon fibre column filtration to give a non adherent (column passed) population and an adherent population recovered from the column. Non adherent, adherent and control (unseparated) populations were tested for cytotoxic activity under the 3 test conditions. Fig. 6 shows the results of a typical experiment using K562 target cells. Nylon-fibre column filtration gave a non adherent population with cytotoxic activity equal to or greater than the control population and an adherent population with lower activity. All 3 populations showed increased activity with IF treatment, and again pretreatment of effector cells produced a larger effect than adding IF to the test.

Using Molt-4 and Bri8 targets, a similar pattern of results was obtained, but with lower levels of cytotoxicity against the latter (Table III). Non-adherent cells showed predictably greater cytotoxic activity than adherent cells but IF treatment considerably increased the reactivity in all effector populations.

Enhancement ratios calculated for each population, as described above, were again comparable, at least for data on K562 and Molt 4.

TABLE III.—*The effect of interferon treatment on cytotoxicity of K562 target cells by blood lymphocyte preparations separated by nylon fibre column filtration*

| Effector population | Interferon treatment (250 u/ml) | Lytic units/$10^6$ cells* — Experiment 1: K562 | Experiment 1: Molt 4 | Experiment 2: K562 | Experiment 2: Bri8 |
|---|---|---|---|---|---|
| Unseparated | None | 11·6 | 14·7 | 8·4 | < 1·0 |
| | Pretreatment (18 h) | 25·0 | 20·8 | 20·8 | 7·7 |
| | In test | 26·3 | 19·2 | 20·4 | 1·6 |
| Non-adherent | None | 12·2 | 21·3 | 15·9 | < 1·0 |
| | Pretreatment (18 h) | 25·6 | 25·6 | 29·4 | 7·1 |
| | In test | 23·8 | 24·4 | 24·4 | 3·3 |
| Adherent | None | 7·9 | NT | 5·1 | < 1·0 |
| | Pretreatment (18 h) | 20·4 | NT | 12·3 | 1·3 |
| | In test | 18·5 | NT | 12·2 | 1·4 |

* Required for 30% cytotoxicity.
NT = Not tested.

### DISCUSSION

The release of viral inhibitors in the supernatants of mixed cultures of lymphocytes and certain tumour-derived or virus-transformed cell cultures was recently described by Trinchieri and colleagues (Trinchieri *et al.*, 1978; Trinchieri & Santoli, 1978) who characterized them as interferons. Two additional properties were associated with these supernatants, which correlated with their antiviral activity; anticellular activity and a capacity to enhance spontaneous lymphocyte cytotoxicity. Since such mixed cultures furnish a plethora of biologically active substances, it was not possible to conclude definitively whether the various activities were mediated by identical or different molecules. As far as enhancement of lymphocyte cytotoxicity was concerned, the probability that IF was the active principle was subsequently strengthened by Herberman *et al.* (1979) and Zarling *et al.* (1979) using purified preparations of lymphoblastoid cell and fibroblast origins, the latter having the advantage over preparations derived from virus-infected or antigen stimulated lymphocytes of lacking virus products and lymphokines. We have also now shown that partially purified human lymphoblastoid IF, produced by Namalva cells, enhances several fold the spontaneous cytotoxicity of human peripheral blood lymphocytes, not only against susceptible, but also against refractory *in vitro* targets. The close correlation of cytotoxicity with interferon titre for the 3 preparations, differing as much as 150-fold in specific activity, provides further corroborative evidence that IF, as distinct from some other lymphokine, is the active agent in these experiments.*

The cytotoxic cells showing activity enhanced by IF were characterized by comparison of the activity of lymphocytes separated by two techniques: adherence to nylon fibre columns and formation of SRBC rosettes. The demonstration that the activity of the IF stimulated and unstimulated cells were similarly distributed strongly suggested that the same cells were involved in both cytotoxic phenomena. We have previously shown that cells with spontaneous cytotoxic activity are heterogeneous, since their activity is present in SRBC-rosette-forming and non-rosette-forming fractions and nylon-fibre adherent and non-adherent fractions (Potter & Moore, 1979). The action of IF would thus appear to be not the generation of a new population of effectors, but an increase in the activity of pre-existing NK cells (Saksela *et al.*, 1979) manifest against all the cell lines tested, whether of

* In this context, it is pertinent to note that both crude and partially purified Namalva IF have no migration inhibitory factor (MIF) activity (Fantes, K.H., personal communication).

high or low susceptibility to unstimulated NK cells.

Enhancement of cytotoxicity was most apparent when effectors were pre-treated with IF and washed before addition to the test. The response was virtually immediate, enhanced cytotoxicity being easily detectable after 1 h and maximal after 6 h. The rapidity of this interaction excludes proliferation of a clone of NK cells as an explanation of our findings, and corroborates our interpretation that the action of IF is to amplify the activity of pre-existing NK cells or their precursors.

The activity of IF-pre-treated effectors also shows that IF probably does not act by providing a recognition–binding system (as is the function of IgG molecules in the induction of antibody-dependent cellular cytotoxicity) but directly stimulates effector cells. The mechanism whereby IF augments NK activity is not known and it remains to be determined whether the multiple effects ascribed above to IF are mediated by a common pathway.

The efficacy of NK stimulation by IF was also reflected in the kinetics of cytotoxicity, where stimulated effectors exerted their lytic activity earlier and more strongly than their untreated counterparts. The high levels of cytotoxicity achieved after 18 h by control effectors in the absence of exogenous IF may be related to the endogenous generation of IF by lymphocytes exposed to K562 cells (Fantes & Moore, unpublished; *cf.* Trinchieri *et al.*, 1977).

Addition of IF to effector:target cell mixtures, as distinct from pre-treatment of effectors alone, also increased cytotoxicity, but to a lesser extent. Reduced availability of IF to effectors, leading to sub-optimal stimulation on account of competitive interaction with targets, is possible, if unlikely, at the E:T ratios used. The probable explanation is that IF exerts an antagonistic effect on targets. Indeed, IF-pretreated targets are more resistant than their untreated counterparts to lysis by both IF-treated and untreated NK cells (unpublished observations). This accords with the data of Trinchieri & Santoli (1978) on certain targets, when an indirect intracellular mechanism was postulated involving synthesis by the cells of both RNA and protein.

Other conditions under which NK activity is augmented *in vitro* may be relevant to our findings. Several studies have shown that human lymphocytes kill virus-infected targets more efficiently than their uninfected counterparts (Santoli *et al.*, 1978*a*, *b*). The high level of IF produced in these systems is held to be the major factor responsible for the increase in susceptibility. During the generation of cytotoxic T lymphocytes in mixed culture by allogeneic lymphocytes, B-cell lines and tumour cells (Peter *et al.*, 1975; Jondal & Targan, 1978; Koide & Takasugi, 1978) there is a concurrent increase in the level of natural cytotoxicity. This is probably caused by the product of an activated lymphocyte (or macrophage) since supernatants from such activated cultures augment NK activity in unstimulated lymphocytes. In some of these studies the kinetics of generation of cytotoxic cells resembles that obtained on IF stimulation. These phenomena could thus conceivably be mediated, at least in part, by endogenous IF, which, among other activated lymphocyte products, is present in such fluids.

It has been argued that interferon, as a potent stimulator of natural cytotoxicity, might render the NK system an inducible selective defence mechanism against transformed and virus-infected cells (Trinchieri & Santoli, 1978). Both types of cell have been shown to induce endogenous IF production on contact with host lymphocytes *in vitro* (Trinchieri *et al.*, 1977) and this interaction could also be a mechanism whereby NK activity is enhanced *in vivo*. Generation of IF as an important factor in host defences has recently gained support from, among other studies, animal experiments in which different anti-tumour agents putatively acting *via* the common pathway of IF induction have been shown to augment NK activity *in vivo* (Oehler *et al.*, 1978). That IF augments NK activity in man was recently shown by Huddlestone *et al.* (1979) in studies of patients with non-Hodgkin's lymphoma.

In this context the demonstration that IF induces cytotoxicity against cells which are refractory to lysis by unstimulated effectors is potentially significant. However, in our view, other issues are also crucial to this hypothesis, including: (i) that the documented antagonism of IF toward target cells does not nullify the enhanced cytotoxicity; and (ii) that spontaneously arising malignant cells that have not been adapted to tissue culture may be lysed by either native or activated NK cells. To these questions, subsequent reports from this laboratory will be addressed (Vose & Moore, 1980).

This study was supported by grants from the Medical Research Council and the Cancer Research Campaign of Great Britain. Interferon was the generous gift of Dr K. H. Fantes, Wellcome Research Laboratories, Beckenham, Kent. We thank Miss Wendy White for skilled technical assistance.

### REFERENCES

ANDERSSON, L. C., NILSSON, K. & GAHMBERG, C. G. (1979) K-562—a human erythroleukaemic cell line. *Int. J. Cancer*, **23**, 143.

BALKWILL, F., WATLING, D. & TAYLOR-PAPADIMITRIOU (1978) Inhibition by lymphoblastoid interferon of growth of cells derived from the human breast. *Int. J. Cancer*, **22**, 258.

BRAUN, W. & LEVY, H. B. (1972) Interferon preparations as modifiers of the immune response. *Proc. Soc. Exp. Biol. Med.*, **141**, 769.

DJEU, J. Y., HEINBAUGH, J. A., HOLDEN, H. T. & HERBERMAN, R. B. (1979) Augmentation of mouse natural killer cell activity by interferon and interferon inducers. *J. Immunol.*, **122**, 175.

DONAHOE, R. M. & HUANG, K. Y. (1976) Interferon preparations enhance phagocytosis *in vivo*. *Infect. Immunol.*, **13**, 1250.

EINHORN, S., BLOMGREN, H. & STRANDER, H. (1978) Interferon and spontaneous cytotoxicity in man. I. Enhancement of the spontaneous cytotoxicity of peripheral lymphocytes by human leukocyte interferon. *Int. J. Cancer*, **22**, 405.

FOLEY, G. E., LAZARUS, H., FARBER, S., UZMAN, B. J., BOONE, B. A. & MCCARTHY, E. (1965) Continuous culture of human lymphoblasts from peripheral blood of a child with acute leukaemia. *Cancer*, **18**, 522.

GISLER, R. H., LINDAHL, P. & GRESSER, I. (1974) Effect of interferon on antibody synthesis *in vitro*. *J. Immunol.*, **113**, 438.

GRESSER, I. (1977) Commentary on the varied biologic effects of interferon. *Cell Immunol.*, **34**, 406.

HAVELL, E. A., YIP, Y. K. & VILCEK, J. (1977) Characteristics of human lymphoblastoid (Namalva) interferon. *J. Gen. Virol.*, **38**, 51.

HERBERMAN, R. B., ORTALDO, J. R. & BONNARD, G. D. (1979) Augmentation by interferon of human natural and antibody-dependent cell-mediated cytotoxicity. *Nature*, **277**, 221.

HERON, Z., BERG, K. & CANTELL, K. (1976) Regulatory effect of interferon on T cells *in vitro*. *J. Immunol.*, **117**, 1370.

HUANG, K., DONAHOE, R. M., GORDON, F. B. & DRESSER, H. R. (1971) Enhancement of phagocytosis by interferon-containing preparations. *Infect. Immunol.*, **4**, 581.

HUBER, C., SUNDSTROM, C., NILSSON, K. & WIGZELL H. (1976) Surface receptors on human haematopoietic cell lines. *Clin. Exp. Immunol.*, **25**, 367.

HUDDLESTONE, J. R., MERIGAN, T. C., JR & OLDSTONE, M. B. A. (1979) Induction and kinetics of natural killer cells in humans following interferon therapy. *Nature*, **282**, 417.

ISAACS, A. & LINDENMANN, J. (1957) Virus interference I. Interferon. *Proc. R. Soc. Lond.* (*Biol.*), **147**, 258.

JOHNSON, H. M., SMITH, B. G. & BARON, S. (1975) Inhibition of the primary *in vitro* antibody response by interferon preparations. *J. Immunol.* **114**, 403.

JONDAL, M. & TARGAN, S. (1978) *In vitro* induction of cytotoxic effector cells with spontaneous killer cell specificity. *J. Exp. Med.*, **148**, 1621.

KOIDE, Y. & TAKASUGI, M. (1978) Augmentation of human natural cell-mediated cytotoxicity by a soluble factor. I. Production of N-cell activating factor (NAF). *J. Immunol.*, **121**, 872.

LINDAHL, P., LEARY, P. & GRESSER, I. (1972) Enhancement by interferon of the specific cytotoxicity of sensitized lymphocytes. *Proc. Natl Acad. Sci.*, **69**, 721.

LINDAHL-MAGNUSSON, P., LEARY, P. & GRESSER, I. (1972) Interferon inhibits DNA synthesis induced in mouse lymphocyte suspensions by phytohaemagglutinin or by allogeneic cells. *Nature* (*New Biol.*), **237**, 120.

MINOWADA, J., OHNUMA, T. & MOORE, G. E. (1972) Brief communication. Rosette forming human lymphoid cell lines. I. Establishment and evidence for origin of thymus-derived lymphocytes. *J. Natl Cancer Inst.*, **49**, 891.

OEHLER, J. L., LINDSAY, L. R., NUNN, M. E., HOLDEN, H. T. & HERBERMAN, R. B. (1978) Natural cell-mediated cytotoxicity in rats. II. *In vivo* augmentation of NK-cell activity. *Int. J. Cancer*, **21**, 210.

PETER, H. H., EIFE, R. F. & KALDEN, J. R. (1975) Spontaneous cytotoxicity (SCMC) of normal human lymphocytes against a human melanoma cell line: A phenomenon due to a lymphotoxin-like mediator. *J. Immunol.*, **116**, 342.

POTTER, M. R. & MOORE, M. (1975) PHA stimulation of separated human lymphocyte populations. *Clin. Exp. Immunol.*, **21**, 456.

POTTER, M. R. & MOORE, M. (1979) Natural cytotoxic reactivity of human lymphocyte subpopulations. *Immunology*, **37**, 187.

SAKSELA, E., TIMONEN, T. & CANTELL, K. (1979) Human natural killer cell activity is augmented by interferon *via* recruitment of "Pre-NK" cells. *Scand. J. Immunol.*, **10**, 257.

SANTOLI, D., TRINCHIERI, G. & KOPROWSKI, H. (1978*b*) Cell-mediated cytotoxicity against virus-infected target cells in humans. II. Interferon induction and activation of Natural Killer Cells. *J. Immunol.*, **121**, 532.

SANTOLI, D., TRINCHIERI, G. & LIEF, F. S. (1978*a*) Cell-mediated cytotoxicity against virus-infected target cells in humans. I. Characterisation of the effector lymphocyte. *J. Immunol.*, **121**, 526.

SCHULTZ, R. M., PAPAMATHEAKIS, J. D. & CHIRIGOS, M. A. (1977) Interferon: An inducer of macrophage activation by polyanions. *Science*, **197**, 674.

SENIK, A., GRESSER, I., MAURY, C., GIDLUND, M., ORN, A. & WIGZELL, H. (1979) Enhancement by interferon of natural killer cell activity in mice. *Cell Immunol.*, **44**, 186.

SONNENFELD, G., MANDEL, A. D. & MERIGAN, T. C. (1977) The immunosuppressive effect of Type II mouse interferon preparations on antibody production. *Cell Immunol.*, **34**, 193.

STRANDER, H., MOGENSEN, K. E. & CANTELL, K. (1975) Production of human lymphoblastoid interferon. *J. Clin. Microbiol.*, **1**, 116.

STEWART, W. E., GRESSER, I., TOVEY M. G., BANDU, M. T. & LEGOFF, S. (1976) Identification of the cell multiplication inhibitory factors in interferon preparations as interferons. *Nature*, **262**, 300.

TRINCHIERI, G. & SANTOLI, D. (1978) Anti-viral activity induced by culturing lymphocytes with tumour-derived or virus-transformed cells. Enhancement of human natural killer cell activity by Interferon and antagonistic inhibition of susceptibility of target cells to lysis. *J. Exp. Med.*, **147**, 1314.

TRINCHIERI, G., SANTOLI, D., DEE, R. R. & KNOWLES, B. B. (1978) Anti-viral activity induced by culturing lymphocytes with tumor-derived or virus-transformed cells. Identification of the anti-viral activity as interferon and characterisation of the human effector lymphocyte subpopulation. *J. Exp. Med.*, **147**, 1299.

TRINCHIERI, G., SANTOLI, D. & KNOWLES, B. B. (1977) Tumour cell lines induce interferon in human lymphocytes. *Nature*, **270**, 611.

VIGNAUX, F. & GRESSER, I. (1977) Differential effects of interferon on the expression of H-2K, H-2D and Ia antigens on mouse lymphocytes. *J. Immunol.*, **118**, 721.

VOSE, B. M. & MOORE, M. (1980) Natural cytotoxicity in man: low susceptibility of freshly isolated tumour cells to lysis. *J. Natl. Cancer Inst.* (in press).

ZARLING, J. M., SOSMAN, J., ESKRA, L., BORDEN, E. C., HOROSZEWICZ, J. S. & CARTER, W. A. (1978) Enhancement of T cell cytotoxic responses by purified human fibroblast interferon. *J. Immunol.*, **121**, 2002.

ZARLING, J. M., ESKRA, L., BORDEN, E. C., HOROSZEWICZ, J. & CARTER, W. A. (1979, Activation of human natural killer cells cytotoxic fo human leukaemia cells by purified interferon. *Jr Immunol.*, **123**, 63.